# Systems biology predicts that fibrosis in tuberculous granulomas may arise through macrophage-to-myofibroblast transformation

**DOI:** 10.1371/journal.pcbi.1008520

**Published:** 2020-12-28

**Authors:** Stephanie Evans, J. Russell Butler, Joshua T. Mattila, Denise E. Kirschner

**Affiliations:** 1 Microbiology and Immunology, School of Medicine, University of Michigan Medical School, Ann Arbor, Michigan, United States of America; 2 Division of Biological Sciences, Advent Health University, Orlando, Florida, United States of America; 3 Department of Infectious Disease and Microbiology, Graduate School of Public Health, University of Pittsburgh, Pennsylvania, United States of America; 4 Center for Vaccine Research, University of Pittsburgh, Pittsburgh, Pennsylvania, United States of America; University of California Irvine, UNITED STATES

## Abstract

*Mycobacterium tuberculosis* (*Mtb*) infection causes tuberculosis (TB), a disease characterized by development of granulomas. Granulomas consist of activated immune cells that cluster together to limit bacterial growth and restrict dissemination. Control of the TB epidemic has been limited by lengthy drug regimens, antibiotic resistance, and lack of a robustly efficacious vaccine. Fibrosis commonly occurs during treatment and is associated with both positive and negative disease outcomes in TB but little is known about the processes that initiate fibrosis in granulomas. Human and nonhuman primate granulomas undergoing fibrosis can have spindle-shaped macrophages with fibroblast-like morphologies suggesting a relationship between macrophages, fibroblasts, and granuloma fibrosis. This relationship has been difficult to investigate because of the limited availability of human pathology samples, the time scale involved in human TB, and overlap between fibroblast and myeloid cell markers in tissues. To better understand the origins of fibrosis in TB, we used a computational model of TB granuloma biology to identify factors that drive fibrosis over the course of local disease progression. We validated the model with granulomas from nonhuman primates to delineate myeloid cells and lung-resident fibroblasts. Our results suggest that peripheral granuloma fibrosis, which is commonly observed, can arise through macrophage-to-myofibroblast transformation (MMT). Further, we hypothesize that MMT is induced in M1 macrophages through a sequential combination of inflammatory and anti-inflammatory signaling in granuloma macrophages. We predict that MMT may be a mechanism underlying granuloma-associated fibrosis and warrants further investigation into myeloid cells as drivers of fibrotic disease.

## Author summary

Tuberculosis is a disease caused by *Mycobacterium tuberculosis* (*Mtb*), a bacterium that infects over a third of the world’s population. The only available vaccine for TB has limited efficacy and drug treatment involves several antibiotics that are taken for several months. These drugs can have significant side-effects and a lack of compliance can lead to drug resistance in *Mtb*. A hallmark of *Mtb* infection is the development of clusters of cells that form around infected macrophages to contain the infection called granulomas. Older granulomas, or granulomas in patients treated with antibiotic, often become fibrotic and this can cause chronic lung problems long after the *Mtb* infection has cleared. The process that drives a fibrotic outcome has been difficult to assess *in vivo*. Herein we combined wet-lab and computational experimentation to identify a novel mechanism leading to peripheral fibrosis in granulomas. We find that macrophages transforming into myofibroblast-like cells, may be a key pathway to granuloma-associated fibrosis, a phenomena that has not been well characterized *in vivo*. Further we identified factors that can inhibit fibrosis development during TB that could be therapeutic targets during TB treatment to limit the risk of long-term tissue damage in TB.

## Introduction

**Fig 1:**
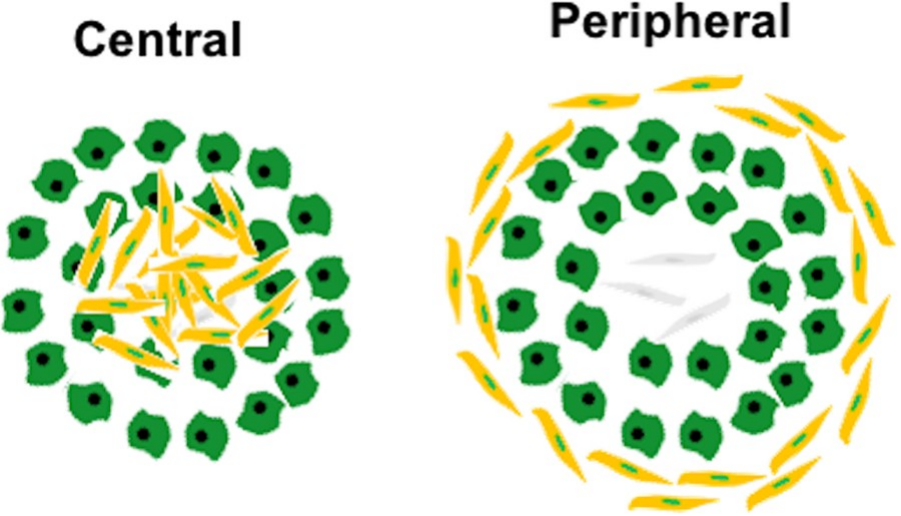
Schematic diagram of the two main organizational types of granuloma-associated fibrosis in TB granulomas indicating the two main types of fibrosis observed in NHP granulomas. Yellow cells represent myofibroblasts, green represent macrophages, and grey fibroblasts.

Tuberculosis (TB) is a deadly infectious disease caused by inhalation of the bacterium *Mycobacterium tuberculosis* (*Mtb*). Control of the global TB epidemic has been limited by many factors including a complicated drug regimen, development of antibiotic resistance, and the absence of a fully protective vaccine against infection and disease [1,2]. Granuloma formation is the hallmark of pulmonary *Mtb* infection and these lung lesions are composed of immune cells that exist in organized microenvironments around mycobacteria-infected cells. Granulomas serve as staging areas for immune responses that kill bacteria and limit their dissemination, but because they are also rich in potential *Mtb* host cells, they are niches for bacterial persistence and replication [3–5]. Fibrosis can occur in granulomas, especially older granulomas from chronic or resolving disease, but fibrosis is especially prominent after antibiotic treatment for *Mtb* infection [4,6,7]. Fibrocalcific lesions are generally better at controlling bacteria and have lower bacterial burdens than those without fibrosis [4]. Fibrosis in TB granulomas is observed in two forms: peripheral and central, with the peripheral form of fibrosis most commonly observed [6] (Fig 1). Paradoxically, although drug-treated granulomas are often fibrotic, granuloma fibrosis presents a major challenge for drug treatment because it can wall off both mycobacteria and infected cells from the action of the immune system and may limit the diffusion of drugs into areas containing mycobacteria [8]. Moreover, fibrotic resolution of disease can leave scar tissue that inhibits full recovery of lung function [9]; thus, identifying how fibrosis arises in TB may lead to treatments that improve outcomes at multiple points of TB interventions.

The mechanisms that promote fibrosis during *Mtb* infection and granuloma development have been difficult to identify in the context of TB. This problem is compounded by the biology of TB in humans where granulomas form in lungs, which cannot be easily sampled, and on a more basic level, there are challenges associated with a lack of consensus regarding how to identify and characterize human fibroblasts in tissues [10,11]. In studies using cynomolgus macaques, a nonhuman primate (NHP) that closely mimics human TB [12,13], the cytokines IL-4, IL-13, and TGFβ have been implicated in collagen-I expression in the outer cuff of granulomas [6,14,15]. Idiopathic Pulmonary Fibrosis (IPF), a condition characterized by spontaneous and pathologic development of fibrosis in lungs, can offer insights in this area. Lung resident fibroblasts are the primary cell type implicated in IPF pathogenesis [16]; however, there is evidence that myeloid derived cells may also play a role in collagen deposition [17,18]. Circulating fibrocytes in the peripheral blood, have also been implicated as contributors to fibrosis in both liver and lung [19,20]. Further, epithelial–mesenchymal transition (EMT) and macrophage-myofibroblast transition (MMT) have been identified as a source of collagen producing cells in fibrosis. These data suggest that granuloma fibrosis likely requires interaction between multiple cell types at currently unknown temporal and spatial scales, and a deeper understanding of these dynamic processes is needed.

**Fig 2:**
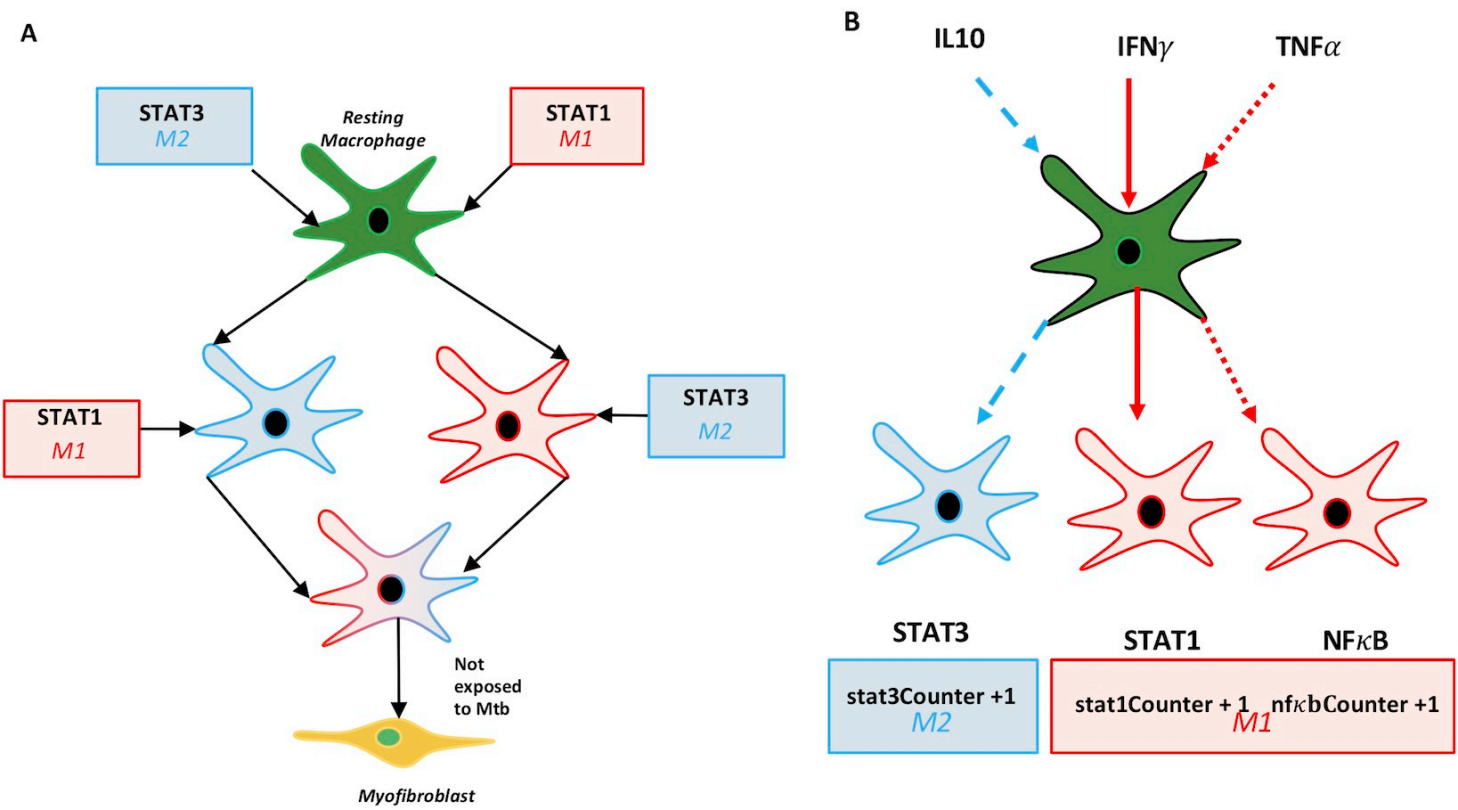
Macrophage polarization and differentiation pathways in *GranSim*. A: Representation of signaling pathways for regulatory macrophages and classification of signals as M1 (inflammatory) or M2 (anti-inflammatory). Every time a macrophage receives a signal on the specific pathway a counter is incremented representing the total stimulation level (e.g. stat1Counter). B: Schematic diagram of criteria for macrophage to myofibroblast transformation (MMT) that were implemented in *GranSim* following, namely that resting macrophages that have STAT1 and STAT3 signals and are not exposed to *Mtb*.

Previous work from our group explored the role of fibroblast to myofibroblast differentiation in granuloma fibrosis [21]. These results demonstrated that the transition of lung-resident fibroblasts to myofibroblasts resulted in a type of fibrosis that was not restricted to granulomas but affected the entire lung environment. However, imaging studies have shown that fibrosis developing as a result of *Mtb* infection can be tightly associated with granulomas, and it is also common for fibrosis to be present only on the periphery of granulomas [6]. There is evidence that fibroblasts or fibroblast-like cells contribute to granuloma-associated fibrosis in NHPs in the absence of IL-10 [22]. However, questions remain as to whether resident fibroblasts are the sole cell type responsible for the development of peripheral granuloma fibrosis or if other cell types are involved through processes such as macrophage to myofibroblast transformation (MMT; Fig 2A). To provide insight into the origins of fibrosis in TB granulomas, we paired computational modeling with wet lab studies and predict a new hypothesis for the mechanisms leading to the development of peripheral, granuloma-associated fibrosis. This fibrotic phenotype is commonly observed, and likely to have important implications for TB pathophysiology, and has not previously been captured with our computational model.

To generate our hypotheses we used *GranSim*, our next-generation computational model, to explore a possible role for MMT in fibrosis at the lesion level, and then classified cells in the fibrotic cuff of fibrotic granulomas from non-human primates (NHPs) to identify their origin. The computational model [23], which we continuously curate with the latest biologic datasets and recently have updated to include fibroblast/myofibroblast differentiation [21] and macrophage polarization dynamics [24] allow us to assess potential spatio-temporal factors underlying granuloma development. Our simulations suggest there is a role for MMT in development of peripheral fibrosis in TB granulomas rather than fibrosis being solely a fibroblast-driven process. We conclude that for MMT to contribute to granuloma-associated fibrosis it must be driven by sequential stimulation of M1 macrophages with an anti-inflammatory signal and inhibited by excessive inflammation/inflammatory signaling and *Mtb* burden. Our results suggest that MMT could occur in granulomas and raise the possibility that this process could be targeted therapeutically to improve resolution of TB and the efficacy of drug treatments.

## Materials and methods

### Ethics statement

The Institutional Animal Care and Use Committee of University of Pittsburgh approved all experiments. The samples came from animals enrolled in completed studies. While in-study, animals were housed and maintained in accordance with standards established in the Animal Welfare Act and the Guide for the Care and Use of Laboratory Animals. The University of Pittsburgh is an AAALAS certified program. **Non-human primate studies:** Formalin-fixed paraffin-embedded (FFPE) tissue sections were cut from samples obtained from macaques as previously described [4,7]. Antigen retrieval and staining were performed as previously described [5,22]. Tissue sections were stained for vimentin (chicken polyclonal, 1:100 dilution; Novus Biologicals, Centennial, CO), *α*-smooth muscle actin (*α*SMA) (clone 1A4, 1:100 dilution; ThermoFisher Scientific, Waltham, MA), CD11c (clone 5D11, 1:30 dilution; Leica Microsystems, Buffalo Grove, IL), and CD31 (clone LCI-9, 1:50, Abcam, Cambridge, MA). Coverslips were applied using Prolong Gold mounting medium containing DAPI (ThermoFisher Scientific) and slides were imaged using a Nikon e1000 immunofluorescent microscope (Melville, NY) with a scanning stage. Images were saved as TIFF-format images, and processed with the Fiji build of ImageJ and the Template Matching plugin to align samples [25,26].

### Image analyses

#### Identification of fibroblasts in IHC images

Images of stained granulomas were imported into ArcPro 2.4 (Esri; Redlands, CA), a Geographical Information Systems (GIS) software as we have done previously [27]. These images were enhanced to identify fibroblasts by their staining for *α*SMA expression and morphology. We then used an iso-clustered, unsupervised classification analysis from toolbox of ArcPro2.4 to the images with classification parameters to maximize the spectral and spatial detail in the images. The classes that resulted were collapsed into two categories. First, we labelled those cells that by linear dimensions (size and shape-see below for calculation) and staining for *α*SMA represented fibroblastic structures. Second, all other cell classes were grouped into a separate category called ‘other’. This produced a layer in GIS that we collapsed only to the category of interest (called a raster) that was converted to vector data (polygons). Because structures other than those of interest in the granuloma images also have similar spectral characteristics as fibroblasts (i.e. same color and brightness), we then filtered the images using two additional filters: 1) polygon geometry and 2) length and area. These known fibroblast polygon parameters from control data spatially filtered out non-fibroblast like polygons. This process distinguished fibroblast from non-fibroblast-like structures and their locations within the images. Finally, we used a spatial-selection polygon to further filter-out non-fibroblast polygons, which refined the identification of fibroblasts. From these methods, we identified the size and shape, number and location of fibroblasts in the granuloma images.

#### Granuloma border interpolation

The process of identifying fibroblasts and other cell classes above helped with the identification of granuloma borders. We then used an unsupervised iso-clustering approach from ArcPro 2.4’s toolbox to digitally classify granuloma images into feature classes based on color and brightness of each pixel. This technique resulted in eleven unique classes, representing different cell-types, cellular debris and image background (no cells, spaces). We further combined these eleven feature classes into three feature classes that revealed differences between tissue preparation background, granuloma, and extra-granuloma locations. This combination of three feature classes revealed a narrow (20-100micron), border between granuloma and extra-granuloma cells and structures. We further enhanced granuloma border location by constructing a minimum-bounding polygon around the fibroblast collar.

#### Calculation of length and width of fibroblasts using GIS

Noise arises when classifying an image using GIS, particularly as IHC images are imperfect 2D representations of 3D object. The slices capture parts and pieces of cells in the Z-plane, and thus deriving exact dimensions for all cells is not possible. This is referred to as edge effect. As the cell’s length is long, 1–2 um differences are negligible with large samples sizes. However, width on such a narrow object is greatly affected by edge effect. Therefore, the width is derived from a calculation but the length is exact. As Area = Length x Width, so Width = area /length. It is standard to multiply the quotient by 2 to capture the 3D edge effect.

#### Hybrid multi-scale model (*GranSim*)

In this work we build the macrophage to myofibroblast transition model into our existing hybrid multi-scale agent-based model (ABM) of granuloma formation, *GranSim*. This model has been curated and used for testing hypothesis in TB since 2004. The model has been developed in conjuction with an experimental collaboration with the laboratory of Dr. JoAnne Flynn at the University of Pittsburgh and has been validated against large and numerous datasets regarding the immune response to *M*. *tuberculosis* within lungs of non-human primates (NHP) leading to the formation of granulomas [21,23,28] *(s*ee our detailed website http://malthus.micro.med.umich.edu/GranSim for full model details, all published manuscripts using this model and an executable program). Briefly, *GranSim* tracks the immune response in lungs following infection with *Mtb* that ultimately results in either emergence of a granuloma (if the initial infection is not cleared by the first infected macrophages). *GranSim is an agent-based (individual-based) model (ABM) that is comprised of 5 features*: ***Agents****-*are individually tracked immune cells as follows: four macrophage states (resting, activated, infected, and chronically infected- see below for more details), three T cell classes (cytotoxic, IFNγ producing, and regulatory), fibroblasts and myofibroblasts (see below for more details), as well as cytokines IFN-γ, TGF*β*, IL10, TNF*α*, chemokines CCL2, CXCL9 and CCL5 and individual bacteria in 3 states (intracellular, extracellular and non-replicating trapped with caseum) [21]; ***The Environment***: Or environment represents a section of lung tissue that is 4 mm x 44 mm in size, which allows for simulated granulomas to grow as large as typical in vivo granulomas. The grid is 2D (although we have a 3D version available when needed- see http://malthus.micro.med.umich.edu/3D-GranSim/) and the lung grid is partitioned into 20 micron x 20 micron microcompartments, as that is the typical size of our immune largest immune cells under study, macrophages and fibroblasts. The lung grid also is populated with blood vessel ports that were placed based on studies of healthy NHP lungs where cells, chemokines, or cytokines can enter into the lung space. ***Rules***: Rules are the probablistic interactions between cells and each other as well as the environment. These are based and validated on hundreds of observed interactions of primate immune cells and molecules *in vivo* and both calibrated and validated by extensive NHP datasets;. ***Parameters*:** The model is parameterized by hundreds of parameters that are estimated from data and further studied using uncertainty and sensitivity analyses; Finally, ABMs are defined by the time step of the fastest process which in our case is diffusion of molecules, that runs on a 6-second time scale. The model when simulated, processes rules and agents at the cell and molecular scale and leads to emergent behavior of a single granuloma at the tissue scale.

### Details of Fibroblasts and Myfibroblasts witin the GranSim framework

We have added into the *GranSim* architecture fibroblasts and myofibroblasts as well as TGF-β, a major signaling molecule involved in MMT. We also have studied M1 to M2 macrophages with our model using a sub-model that tracks NFkb and STAT1 signalling within macrophages [24,29–31]. This allowed us to define these macrophage types on a spectrum based on what signals are received, rather than binary. In the work herein, we utilize this previously published model of protein signaling pathways inside of a macrophage to define the signaling profile of macrophages and as an indication of their inflammatory or anti-inflammatory phenotype (Fig 2B). Macrophages in GranSim differentiate into myofibroblasts with a given probability based on discrete conditions (aka *Rules*) in their environment. *Resting* macrophages become activated if they are exposed to IFN-γ, which also programs the macrophage agent to register that it has been STAT1 stimulated, or TNF-α. Resting and *Activated* macrophages become infected if they take up Mtb agents, and *Infected* macrophages become *Chronically Infected* if they contain more than a threshold number of bacteria (about 25, but this is varied in the parameter file). If a macrophage is exposed to IL-10, it downregulates some phagocytic capabilities and becomes STAT3 stimulated. The level of gene stimulation is a macrophages property that allows the model to track each agent’s history; however, it is the real-time environmental conditions that effect the state of a macrophage at each time step. Warsinske *et al*. presented the addition of fibroblasts to GranSim by the inclusion of the following rules: Fibroblasts can divide if they bind a sufficient amount of TGF-β, have not bound more than a maximum amount of IL10, and are within close enough proximity to the granuloma. Fibroblasts can differentiate into myofibroblasts if they have not proliferated in the current time step, have bound a sufficient amount of TGF-β1, have not bound more than a maximum amount of IL10, and are present in a sufficient concentration of TNFα to indicate close proximity to the granuloma. Fibroblasts are motile cells but myofibroblasts remain stationary. To facilitate macrophage to myofibroblast transformation (MMT) in *GranSim* we created a function that forces resting macrophages to irreversibly switch phenotypes and become myofibroblasts immediately if they receive both STAT1 and STAT3 signals through the appropriate pathways while they remain negative for Mtb exposure (Fig 2A). Through our simulation studies discussed at length in Results, we determined the rules for macrophages to transition to myofibroblasts to be as follows: a macrophage must have received M1 polarizing signals followed by M2 polarizing signals.

#### Computational platform

*GranSim* is constructed through use of the C++ programming language, Boost libraries (distributed under the Boost software license; https://www.boost.org), and the Qt framework for visualization (distributed under General Public License). The ABM is cross-platform (Macintosh, Windows, Unix) and runs with or without visualization software. *GranSim* model simulations were performed on the XSEDE Comet supercomputer (see Acknowledgment).

#### Statistical modeling and data analyses

We recorded the location and status of every macrophage in the *GranSim* simulation at five-day intervals and used the radius of the granuloma to determine if a macrophage agent was located in a central or peripheral location within a granuloma. To determine the radius, the granuloma boundary was determined using our previously published algorithm [32]. Macrophage agents located between the granuloma boundary and 0.8*radius away from the boundary were considered to be "peripheral", those outside of the boundary were classed as not within a granuloma (i.e. “not in gran”) and the remaining macrophages were assigned to the “central” group. To identify characteristics that were significantly different between peripheral and central macrophages, we used a *generalized linear model* (GLM) from the stats package in R [33] with a binomial link function to identify macrophage characteristics that were most strongly associated with being on the periphery of a granuloma at days 50 and 100. The characteristics that we included as variables in the GLM, as well as their regression coefficients (estimates), standard errors, z- and p-values are listed in Table 1.

**Table 1:** Parameters used in GLM for predicting the location of a macrophage. Locations were coded as either peripheral (1) or central (0) depending on their proximity to the granuloma boundary (see Methods for more details).

Model: Location ~ Intercept + stat1Counter + stat3Counter + stat1Counter*stat3Counter + exposureToMtb + nfkbCounter					
**Parameter**	Estimate	Std Error	z-value	p-value	
**Intercept**	-0.104	0.021	-4.851	0.000	***
**Stat1Counter (Level of STAT1 Activation)**	0.002	0.003	0.579	0.563	
**Stat3Counter (Level of STAT3 Activation)**	0.175	0.096	1.821	0.069	
**Exposure to Mtb**	-5.554	0.169	-32.951	0.000	***
**Nfkb Counter (Level of NFkB Activation)**	-10.257	61.765	-0.166	0.868	
**Stat1Counter:Stat3Counter**	0.069	0.010	6.701	0.000	***

#### Global Sensitivity Analysis

We used global sensitivity analysis to identify the influential parameters driving MMT in the simulation. Latin Hypercube Sampling (LHS) [34] was used to generate 100 parameter sets varying the parameters over ranges listed in Table 2 over the respective ranges, to analyze epistemic uncertainty. We performed 3 replicates of each parameter set to allow us to observe the degree of stochasticity exhibited by the simulations with each parameter set, to assess aleatory uncertainty. To quantify the relationship between the parameter values and the model output “number of fibroblasts” over time, we calculated partial rank correlation coefficients (PRCCs, values between -1, 1) using the *Spartan* package in *R* [35]. We class PRCCs as significant if the *p*-values are less than 0.05, calculated by a Z-test. A positive PRCC indicates that the parameter is associated with an increase in numbers of fibroblasts while negative PRCCs are parameters are associated with a decrease in the number of fibroblasts. The use of LHS and PRCCs in sensitivity analysis is reviewed in [32,36].

**Table 2:** Parameter names, definitions and ranges used for LHS.

Parameters		PRCC	p-value
Mac: dIL10Act	Secretion rate of IL10 by activated macrophages (mol/sec)	0.174	0.004
Mac: dIL10Inf	Secretion rate of IL10 by infected macrophages (mol/sec)	0.355	0.000
Mac: thresholdNFkBTNF	TNF threshold for NFkB activation	-0.262	0.000
Mac: probKillExtMtbRest	Probability a macrophage will kill an extracellular Mtb	0.004	0.942
Mac: nrExtMtbUptakeAct	Number of Mtb an activated macrophage can uptake	0.134	0.028
Mac: stat1Beta	STAT1 Transcription/Gene Amplification Factor	0.020	0.747
Mac: nfkbBeta	NFkBTranscription/Gene Amplification Factor	0.036	0.554
Mac: stat3Beta	STAT3 Transcription/Gene Amplification Factor	0.055	0.367
Mac: tgfbActivationFraction	Fraction of latent TGFb activated by a macrophage	0.014	0.814
Mac: TGFBmax	Max amount of TGFB for inhibiting bacterial killing by macrophage	-0.114	0.061
Tcell: TGFBmax	Max amount of TGFB for inhibiting t-cell proliferation	-0.093	0.128
Tcell: tgfbBindingRate	Fraction of active TGFB that is bound by a T cell	-0.087	0.156
Treg: dIL10	Secretion rate of IL10 by T cells (mol/sec)	0.645	0.000
Fibroblast: IL10ProlifMax	Threshold for IL10 above which fibroblast will not proliferate	0.003	0.955
Fibroblast: myoTGFBIL10Threshold	Threshold for ratio of TGFb to IL10 above which fibroblast will differentiate into myofibroblast	0.223	0.000
Mac: maxRecProb	Maximum recruitment probability for macrophages	0.169	0.005
Tgam: maxRecProb	Maximum recruitment probability for Tgams	0.015	0.808
Tgam: thresholdRecChemokine	Threshold of chemokine for chemokine-dependent recruitment of gamma-producing T cells	0.005	0.939
Tgam: thresholdRecTNF	Threshold of TNF for TNF-dependent recruitment of gamma-producing T cells	-0.031	0.612
Tgam: recruitmentHalfSatTNF	Half-saturation parameter for TNF-dependent gamma-producing T-cell recruitment	0.083	0.172
Tgam: recruitmentHalfSatChemokine	Half-saturation parameter for chemokine-dependent gamma-producing T-cell recruitment	0.021	0.733
Tgam: probCognate	Probability a recruited gamma-producing T cell is cognate	-0.011	0.862
Tcyt: maxRecProb	Maximum recruitment probability for cytotoxic-T cells	-0.118	0.052
Tcyt: thresholdRecChemokine	Threshold of chemokine for chemokine-dependent recruitment of cytotoxic T cells	0.065	0.287
Tcyt: thresholdRecTNF	Threshold of TNF for TNF-dependent recruitment of cytotoxic T cells	-0.166	0.006
Tcyt: recruitmentHalfSatTNF	Half-saturation parameter for TNF-dependent cytotoxic T- cell recruitment	-0.042	0.492
Tcyt: recruitmentHalfSatChemokine	Half-saturation parameter for chemokine-dependent cytotoxic T-cell recruitment	0.138	0.023
Tcyt: probCognate	Probability a recruited cytotoxic T cell is cognate	0.151	0.013
Treg: thresholdRecChemokine	Threshold of chemokine for chemokine-dependent recruitment of regulatory T cells	-0.015	0.804
Treg: thresholdRecTNF	Threshold of TNF for TNF-dependent recruitment of regulatory T cells	-0.115	0.059
Treg: recruitmentHalfSatTNF	Half-saturation parameter for TNF-dependent regulatory T-cell recruitment	-0.197	0.001
Treg: recruitmentHalfSatChemokine	Half-saturation parameter for chemokine-dependent regulatory T-cell recruitment	-0.018	0.765
Treg: probCognate	Probability a recruited regulatory T cell is cognate	0.638	0.000

## Results

Previous studies from our group demonstrated that including only fibroblasts as a driver of fibrosis results in collagenization that spreads into the lung environment and cannot adequately reproduce the tightly maintained spatial structure of peripheral granuloma-associated fibrosis [21]. Given the evidence regarding a role MMT in other fibrotic diseases, we sought to investigate whether macrophages could play a role in peripheral granuloma-associated fibrosis and what properties these macrophages must have.

### MMT in the virtual granuloma model, driven by STAT1 and STAT3 and inhibited by exposure to *Mtb*, produces spatial patterns of fibrosis analogous to those identified in peripheral and centrally fibrotic granulomas

**Fig 3:**
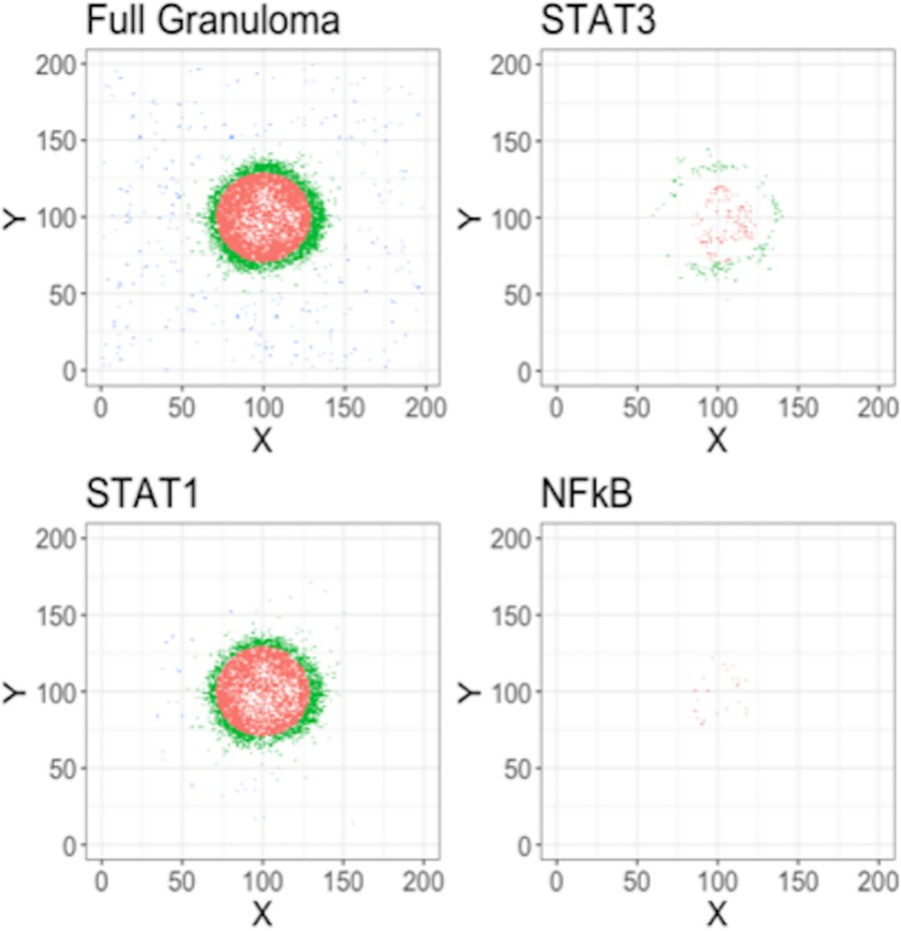
Macrophage locations within simulated granulomas at 50 days. Cells were characterized as “central” if they were located less than 80% of the radius of the granuloma away from center or “peripheral” if they were between 80% and 100% of the radius. Remaining cells were categorized as “Not in granuloma”.

To explore the potential drivers of MMT in fibrosis we recorded the position and status of all the macrophages in our computational granuloma, and classified them as being either peripheral, central, or outside of the granuloma according to their coordinates on the grid, thereby aligning them with types of fibrosis previously identified *in vivo* [6]. Our group has previously published a framework for macrophage signaling pathways that allowed us to count the number of STAT1, STAT3, and NFκB stimulations that a cell has received and assess the signaling profiles of each macrophage category spatially and temporally [36]. Using this model for macrophage status, we identified the key features of macrophages in each of the classified areas. STAT1+ macrophages were present in all three granuloma locations (central to, peripheral to, and outside of the granuloma), STAT3+ macrophages were located within the central and peripheral zones, and NFκB+ cells we found were restricted to the central location as shown via IHC stained NHP granulomas (Fig 3).

**Fig 4:**
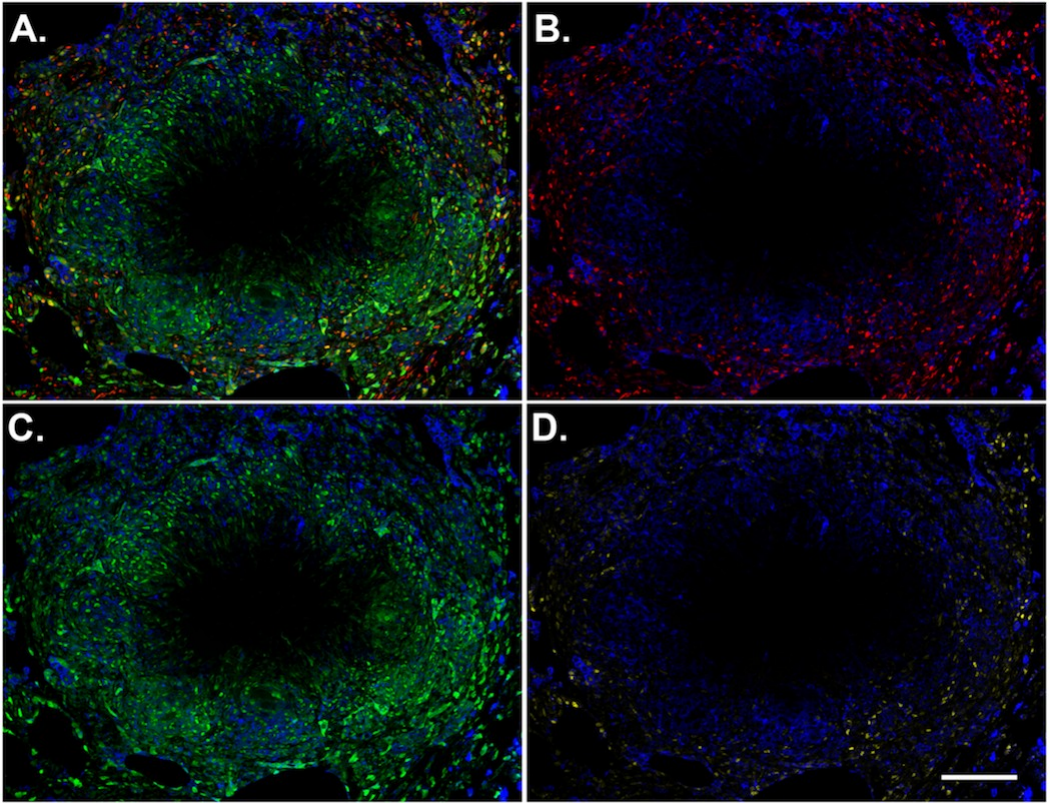
Granulomas have spatially-distinct patterns of STAT1 and STAT3 phosphorylation, including phospho-STAT1+STAT3+ cells. A macaque granuloma was stained for p-STAT1 (green) and p-STAT3 (red) and CD163 (blue). ImageJ was used to separate the (A) merged channels into (B) p-STAT3 and (C) and p-STAT1 channels. (D) ImageJ’s image calculator function was used with the AND command to identify pixels where p-STAT1 and p-STAT3 are colocalized (yellow). CD163 (blue) was included in each panel for context. Scale bar represents 200 μm.

We then used a *generalized linear model* (*GLM*) to identify the macrophage properties (listed in Table 1) that predict the location status of an individual cell (peripheral vs central). We restricted our analysis to timepoints greater than 20 days because evidence suggests that it takes approximately three to four weeks for a granuloma to form [37]. To fit the GLM, we first filtered out all the cells classified as being outside of the granuloma (i.e. “Not in gran”). The macrophage properties that were significantly associated with a macrophage being in a peripheral location included joint STAT1 and STAT3 macrophage stimulation and not being exposed to *Mtb* (*p* < 0.05). We used Latin Hypercube Sampling (LHS) to vary model parameters driving STAT1, STAT3, NFκB, as well as other general granuloma parameters, and performed simulations under each parameter set (3 replications of 100 parameter sets, 300 simulations in total). We used the results from these LHS simulations to identify locations where the MMT-induced fibrosis could occur under criteria of joint STAT1 and STAT3 stimulation as well as non-exposure to *Mtb* in different settings. We then highlighted STAT1/STAT3 simulated macrophages that were not exposed to *Mtb* in images of simulated granulomas to determine where MMT could occur in different granulomas. This analysis revealed that STAT1/STAT3+ *Mtb*- macrophages exhibit spatial distributions analogous with central-, peripheral-, full, and no fibrosis as observed in published imaging studies (Fig 3). To assess whether dual STAT1/STAT3+ activated macrophages are present in granulomas, and were they are located, we used IHC to visualize the phosphorylated versions of these proteins in NHP granulomas. We found spatially-separated STAT1 and STAT3 expression (Fig 4A), with phospho (p)-STAT3+ cells being most abundant in the lymphocyte cuff and granuloma periphery (Fig 4B) and p-STAT1+ cells being most abundant in the epithelioid macrophage region (Fig 4C). When we examined colocalization of these two phosphorylated STAT proteins, we found were substantial numbers of p-STAT1+p-STAT3+ cells in the granulomas, particularly in the granuloma’s lymphocyte cuff and peripheral regions (Fig 4D). These biologic data support our computational findings and indicate that different granuloma regions have unique macrophage polarization states.

**Fig 5:**
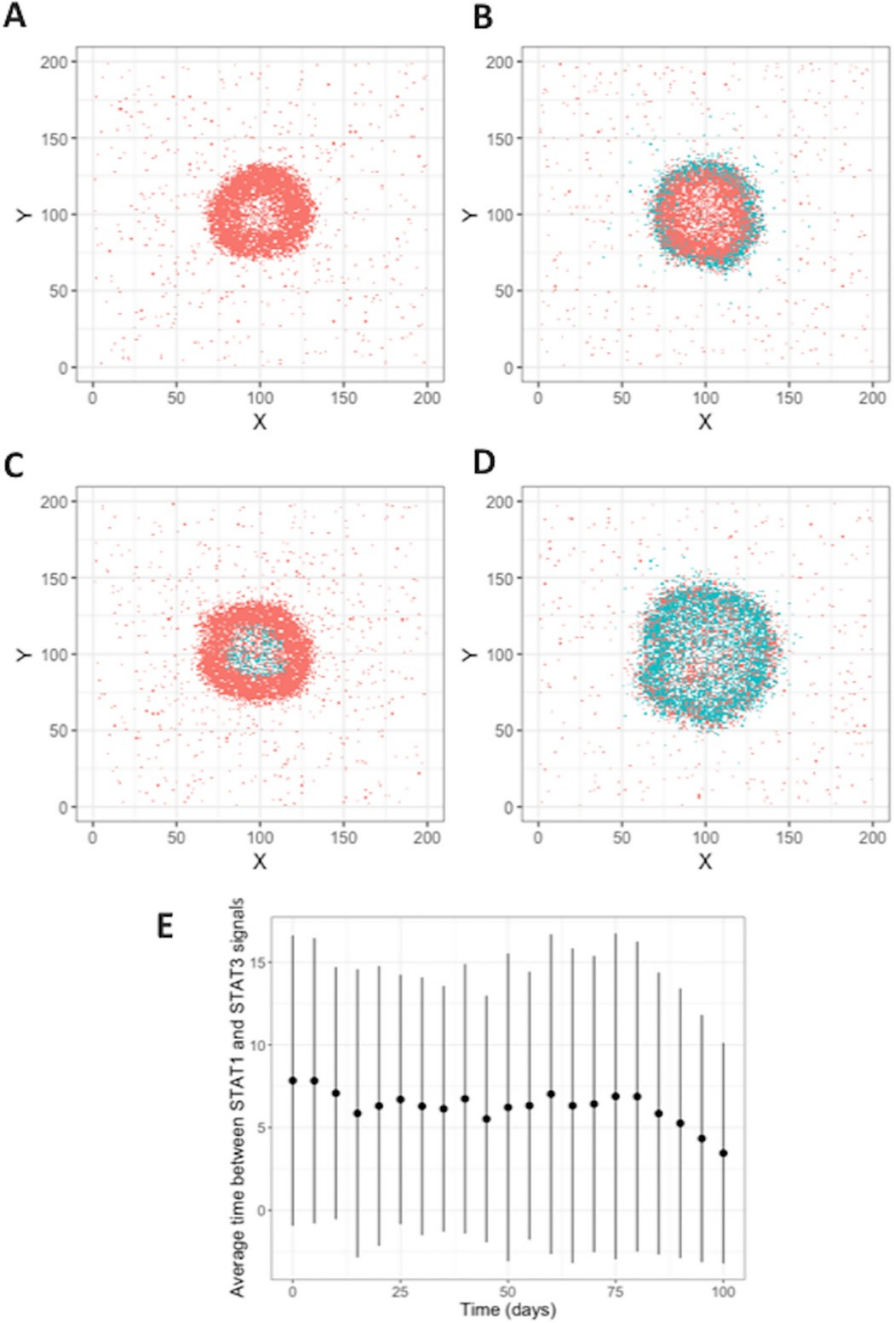
Four distinct types of fibrosis phenotypes produced by the hypothesized MMT rules. Plots show the macrophages that meet the STAT1+STAT3+Exposure- criteria for MMT (turquoise), or not (coral), at 50 days post-infection. The four fibrosis types are A: central fibrosis, B: peripheral fibrosis, C: fully fibrotic, and D: non-fibrotic. E: Average time between STAT1 phosphorylation signals in *GranSim* simulations. STAT1 signal comes before STAT3 signal in macrophages that are candidates for MMT.

Previous research has suggested that macrophages that are able to transition to become fibrotic through MMT are M1 polarized macrophages [38–41] but our IHC-based observations can only represent the state of granuloma cells at a singular moment without revealing their fate as the granuloma’s development progresses. To test whether the granuloma myofibroblasts were derived from macrophages that experienced inflammatory signals (M1-type macrophages) in our computational model (in line with previous research [38–41], we examined when M1 (STAT1 or NFκB) and M2 (STAT3) signaling occurred in those macrophages identified as undergoing MMT (Fig 5A–5D). For macrophages that are candidates for MMT under the criteria described above, resting macrophages should transition into myofibroblasts when STAT1+STAT3+ are not exposed to *Mtb*, but our rules did not specify the order in which these signals need to be received. Our simulations predicted that for macrophages to meet the criteria for MMT, STAT1 (M1) signaling must come first and the average time between the initiation of STAT1 signaling and initiation of STAT3 signaling is 7.23 days. We did observe instances where macrophages in the granuloma periphery underwent STAT3 signaling before STAT1 (as indicated by the negative error bars in Fig 5E) but this was observed in a small subset of macrophages and it is possible that those cells would not transition into fibroblasts but would instead retain their original M2 state. The time between signals is slightly lower after 10 weeks post-infection, likely due to increased expression of in anti-inflammatory cytokines as the infection begins to resolve (Fig 5E).

### MMT results in biologically relevant fibrosis phenotypes

**Fig 6:**
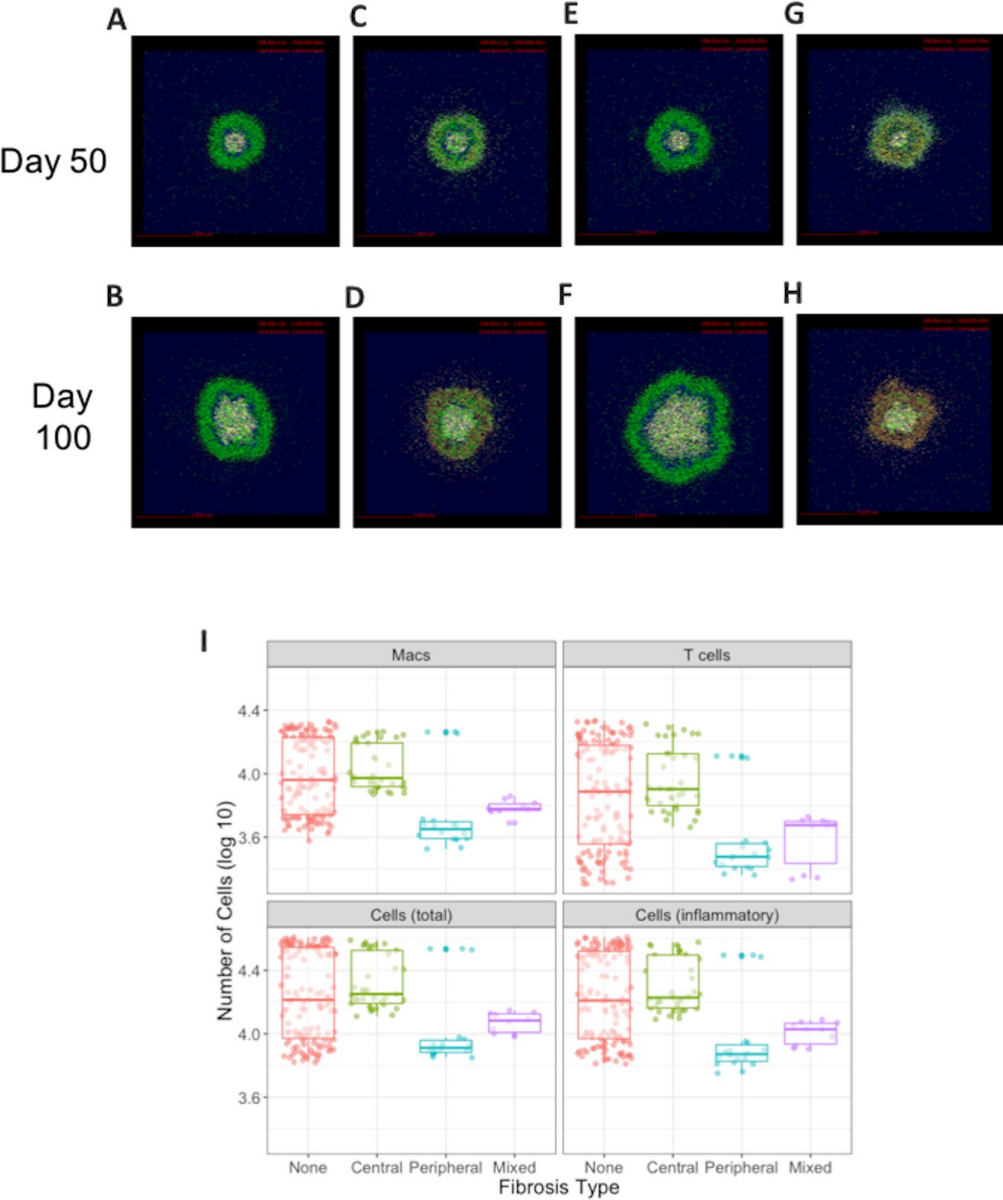
Snapshots of *GranSim* simulations show fibrosis occurring with MMT present. Four different granulomas and fibrosis phenotypes are shown at days 50 and 100 post-infection with *Mtb*. A,B: No fibrosis, C,D: peripheral fibrosis, E,F: central fibrosis, G,H: fully fibrotic. Cells are represented by different colors as follows: resting macrophages are green, activated macrophages are blue, infected macrophages are orange, chronically infected macrophages are red, IFNγ producing T-cells are pink, cytotoxic T cells are violet, regulatory T cells are cyan, fibroblasts are maroon, and myofibroblasts are gold. I: Fibrosis significantly lowers the total cell count in a virtual granuloma. Total cell counts per granuloma snapshot 100 days after the initial *Mtb* infection are shown on a log_10_ scale.

**Fig 7:**
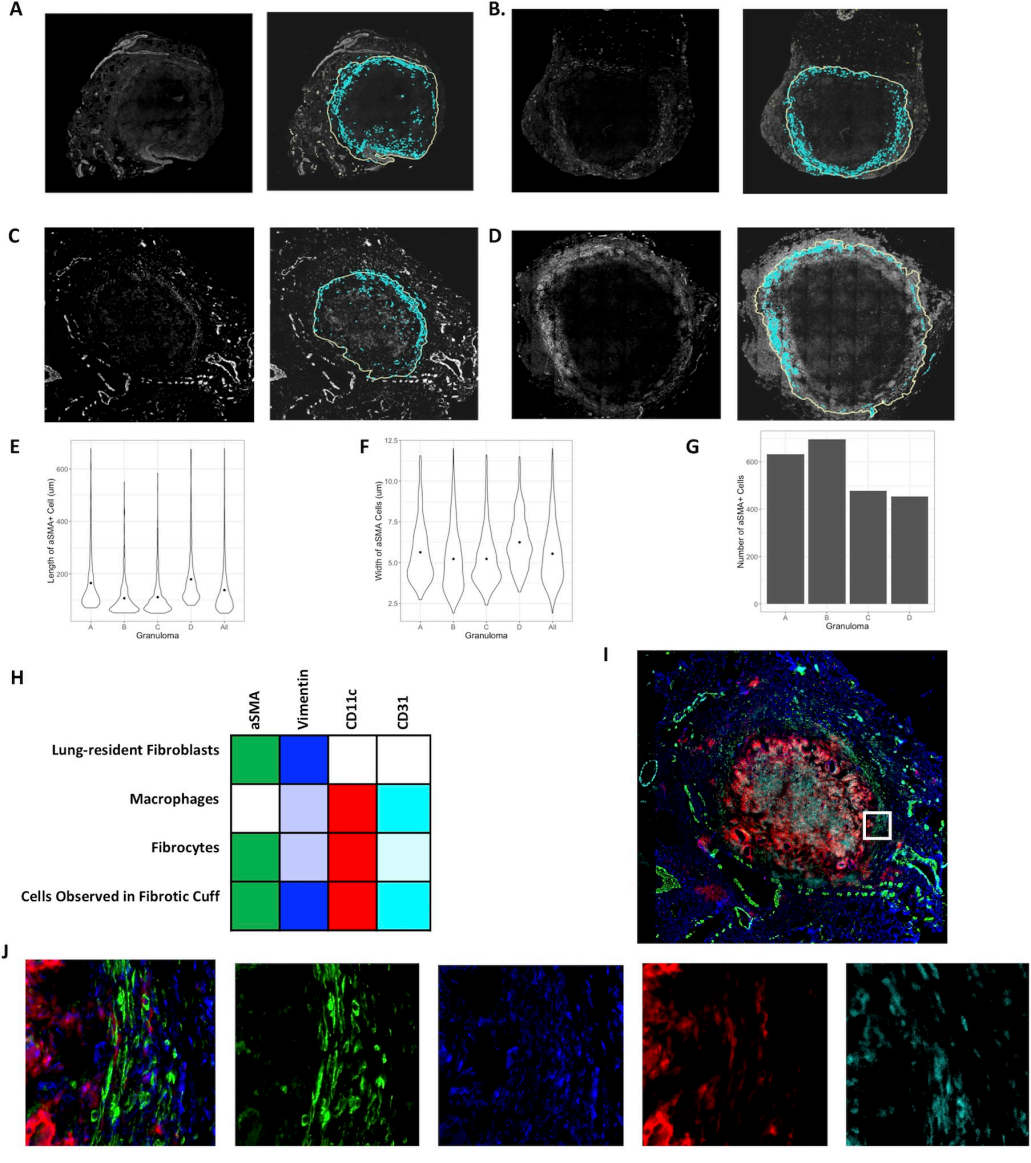
Characterization of fibrosis in the granuloma. A-D: IHC stains for *α* SMA (left panels) and corresponding Geographically Information System (GIS) analysis-identified locations of fibroblasts (right panels) for four unique non-human primate granulomas. Objects identified as fibroblasts are highlighted in cyan, and the granuloma boundary demonstrated by the solid yellow line. E, F: Distribution of lengths (E) and widths (F) of αSMA+ cells for each granuloma respectively. G: Total numbers of αSMA+ cells for each granuloma, respectively. Cells with length or width of greater than three standard deviations away from the mean size were classified as outliers and removed from the plots in E-G.H-J: Representative IHC staining of fibrotic granuloma demonstrate that αSMA (green) expressing cells in the fibrotic cuff co-express CD11c (red), vimentin (blue), and CD31 (cyan). H: Expected expression levels of markers shown in panel I for each cell type involved in fibrosis. Colors correspond to the stains used in B and semi-opaque represents low expression. I: Full staining on sample C. J: Inset, αSMA, vimentin, CD11c, CD31 (left to right).

Our previous results present the spatial distributions of macrophages that are a potential source of MMT in a granuloma. However, since the collagen produced by myofibroblasts alters the granuloma environment, implementing the rules for MMT (i.e. STAT1+STAT3+ non-exposed to *Mtb* macrophages that transition to myofibroblasts) has the potential to alter the observed patterns of fibroblasts arising from MMT from the macrophage locations predicted (Fig 3). To confirm that allowing STAT1/STAT3+ Mtb–macrophages to transition to myofibroblasts does not change the spatial distributions previously observed, we updated the model to include the rules for MMT and ran 3 simulations of 100 parameter sets (300 runs in total). We recorded snapshot images of the simulations at 50 and 100 days, respectively. These snapshots revealed that allowing MMT to occur under the criteria specified results in fibroblast distributions representative of the four fibrotic phenotypes (none, peripheral, central, and full) previously identified Fig 6A–6G). In the 300 simulations from our parameter sets, we considered a granuloma to be fibrotic if more than 450 myofibroblasts were present, a number that is based on the estimated number of myofibroblasts we detected in FFPE NHP granulomas (Fig 7). Under this classification, 33% of the simulated granulomas were fibrotic. We explored the relationship between the phenotype of fibrosis, and the number of cell subsets and total cells per simulated granuloma (Fig 6I). Our results demonstrated that the fibrotic phenotype of a granuloma significantly altered the number of cells remaining in the granuloma environment (one-way ANOVA, p = 0.0003). Granulomas classified as centrally fibrotic generally exhibited higher cell counts than those that were non-fibrotic, peripherally fibrotic, or exhibited mixed fibrosis. However further studies are required to confirm if this result holds true *in vivo* or if it is an artifact of our model system. Those with peripheral or mixed fibrosis had the lowest cell counts after 100 days. There were some outliers in the peripherally fibrotic group that were associated with a less defined ring of fibrosis, although the reason for the lack of definition in the fibrotic ring is unclear.

**Fig 8:**
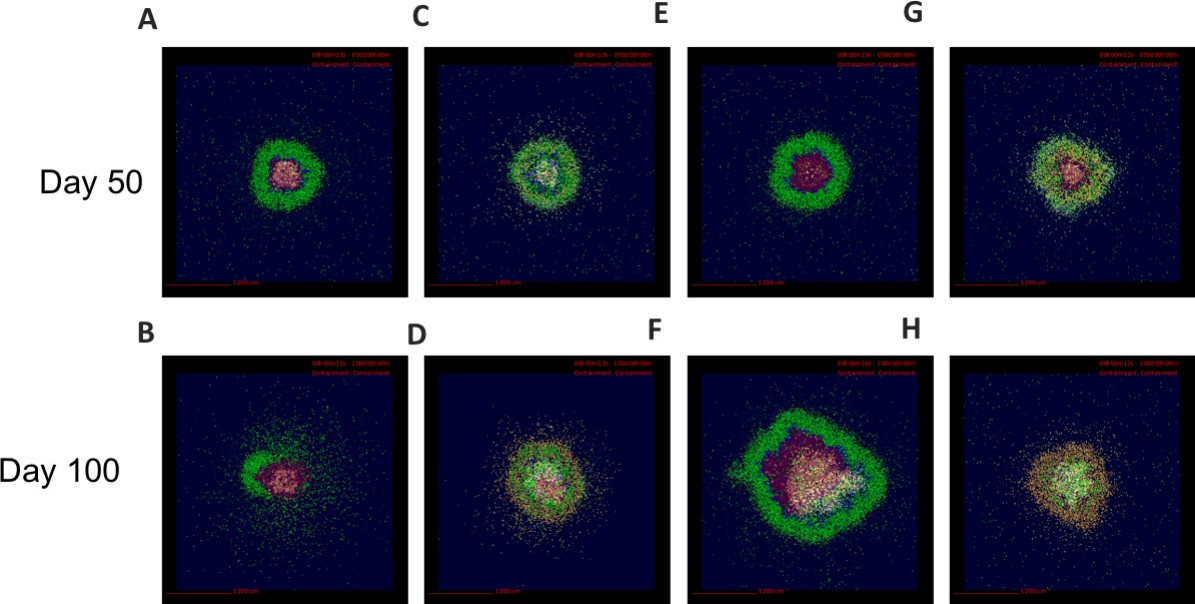
Snapshots of simulation show fibrosis occurring with both MMT and lung-resident fibroblast involvement. A,B: None fibrotic, C,D: peripheral fibrosis, E,F: central fibrosis, G,H: fully fibrotic. Cells are represented by different colors as follows: resting macrophages are green, activated macrophages are blue, infected macrophages are orange, chronically infected macrophages are red, IFNγ producing T cells are pink, cytotoxic T cells are violet, regulatory T cells are cyan, fibroblasts are maroon, and myofibroblasts are gold.

To explore the additional component of interplay between lung-resident fibroblasts and macrophages in granuloma-associated fibrosis, we repeated the simulations in the previous section with the addition of lung resident fibroblasts starting at the time of infection in the model (snapshots are shown in Fig 8). As demonstrated by [21] in the fibroblast alone model, under the combined model of MMT and lung-resident fibroblasts, all of the fibroblasts localized to the center of the granulomas (Fig 8) while the myofibroblasts derived from MMT localized to the periphery, inside of the lymphocyte cuff (S1 Fig). By comparing the snapshots in Fig 6 with those in Fig 8, it is evident that the granulomas that were peripherally and fully fibrotic in the model with MMT alone maintained their structure and phenotype with the addition of lung-resident fibroblasts, but the non-fibrotic granulomas exhibited the beginnings of fibrosis, and the centrally fibrotic granuloma had a higher degree of fibrosis than those granulomas produced by the model MMT alone. This suggests that there is more than one pathway to fibrosis in TB, but also demonstrates that MMT may play an important role in generation of the diverse fibrotic phenotypes observed *in vivo*.

**Fig 9:**
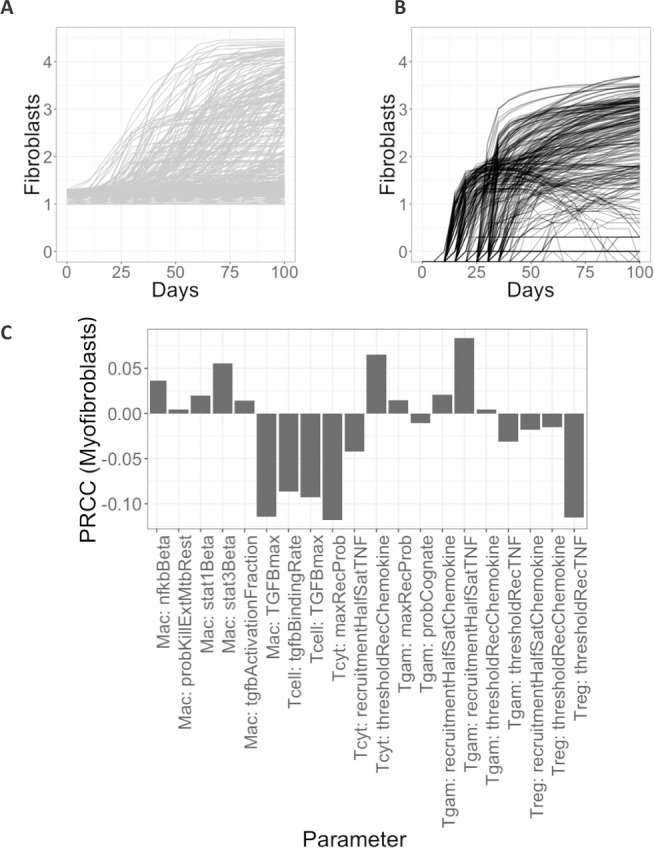
Fibroblast numbers in simulated 2D granulomas (log_10_) comparing where (A) both fibroblasts to myofibroblast differentiation together with MMT or (B) MMT alone. Number of myofibroblasts and activated fibroblasts are shown under A: fibroblasts and MMT involvement in fibrosis, B: MMT alone in fibrosis. Plots are shown on a log base 10 scale. C: Sensitivity analysis reveals key important parameters for increasing the number of myofibroblasts. PRCCs were calculated as described in Methods section and range between -1 and 1. Shown are only the significant PRCC values when the outcome variable is number of myofibroblasts for p < 0.05. Parameters are defined and explained in Table 2.

We can compare the number of fibroblasts over time in a cross section of a virtual granulomas between these two models of fibrosis development (Fig 9A and 9B), and with the estimated number of fibroblasts observed in FFPE granuloma section (Fig 7). In 300 simulations of the model that includes lung-resident fibroblasts in granuloma-associated fibrosis (100 parameter sets, 3 replications, Fig 9A) there are up to 10,000 fibroblasts by 100 days post-infection, almost 100 times higher than the maximum of 600 fibroblasts we observed in the FFPE section (Fig 7). It should be noted that the initial fibroblast counts in under this model are higher than the version with MMT alone since lung-resident fibroblasts are present at the start of the simulation as in [21]. Comparing the counts from FFPE sections to those observed under the model with MMT alone (Fig 9B) demonstrates that this version of the model has more feasible fibroblasts counts ranging from 0 to 1000 (Fig 9A). These data suggest that MMT can generate granuloma-associated fibrosis without the involvement of lung-resident fibroblasts. To further investigate the important parameters that drive MMT in the MMT alone model, we performed a sensitivity analysis on the granuloma-wide parameters that were varied to generate the parameter sets for this study (Table 2 and Fig 9C). The varied parameters were related to the function of T cells and macrophage function and recruitment across all T-cell and macrophage phenotypes, as well as the stimulation pathways for MMT in the simulation.

### The number of fibroblasts is significantly correlated with a healing response

A key strength of modeling is the ability to predict what mechanisms are driving model outcomes. Here, we are interested in identifying what model factors lead to increased numbers of myofibroblasts. To this end, we performed a sensitivity analysis using the LHS/PRCC approach as we have done previously [36]. Our results reveal that the most significant parameters in relation to fibroblast counts in the simulation after 100 days post-infection are related to cytotoxic T cells and regulation by TGFβ (Table 2 and Fig 9C). The probability of a cytotoxic T cell being recruited to the granuloma site is significantly negatively correlated with the number of fibroblasts suggesting that high levels of CD8-type cytokines (for example TNF*α*) in the granuloma reduce the probability of a granuloma becoming fibrotic. Similarly, increasing the amount of TGFβ stimulation required to downregulate a T cell or macrophage also decreases the number of fibroblasts in the simulation. Taken in combination, the sensitivity analysis and PRCCs suggest that fibrosis is a marker of healing in the TB granuloma, and that inflammation inhibits the development of fibrosis. This is in agreement with what is commonly accepted in other disease that fibrosis arises as a dysregulated healing response [42–45].

### Cells in the fibrotic region of TB granulomas co-express CD11c and *α*SMA

To validate our predictions that cells undergoing MMT were M1 macrophages we studied properties of cells in the fibrotic cuff of lung granulomas from NHPs that were necropsied 10–12 weeks post *Mtb* infection and identified key features in relation to what we had observed computationally. The granulomas we selected were scored by a veterinary pathologist as we have done previously [7] and we selected specific lesions with fibrotic cuffs for analysis.

To identify the fibrotic region, these granulomas were stained for alpha-smooth muscle actin (*α*SMA), a marker for myofibroblasts [46]. We found a narrow band of *α*SMA + cells on the periphery of the fibrotic granulomas that is conserved between samples (Fig 7A–7D). The median length and width of *α*SMA+ cells were 108 μm and 5 μm, respectively (Fig 7E and 7F), which is consistent with the ~100 μm length observed for fibroblasts *in vitro* [10,47]. To examine the phenotypes of cells in fibrotic regions, the samples were also stained for vimentin, CD11c, and CD31. The types of cells we expect to be associated with each of the markers, as well as the markers expressed by the cells in the fibrotic cuff are highlighted in Fig 7H. Some cells that we identified as being in the fibrotic cuff of the granuloma slice were only positive for vimentin, CD11c, or αSMA (Fig 7I), however we observed that most of the spindeloid-shaped fibroblasts-like cells in the fibrotic cuff that were positive for CD11c, αSMA, and vimentin (Fig 7I). CD11c+αSMA+vimentin+ cells appeared in all granulomas that we stained suggesting that some of the cells in the fibrotic area of the granuloma are not derived from lung resident fibroblasts [10]. It is possible that these cells could be fibrocytes, fibroblast-like, peripheral blood cells, so to attempt to distinguish these cells from fibrocytes, we also looked at the expression of CD31, which has been reported to be at high levels on some monocytes and macrophages but at low levels on fibrocytes (Fig 7J) [10]. We observed very little difference in the intensity of CD31 in the cells in the fibrotic cuff when compared to the CD11c+ cells in the center of the granuloma, thus we hypothesize that the CD11c+αSMA+cells in the fibrotic cuff are of macrophage origin and that they may arise through the process of macrophage to myofibroblast transformation (MMT). These granulomas showed an overlap of STAT1/STAT3 positive cells on the periphery of the granuloma, where we would expect MMT to occur in a granuloma undergoing a controlling/healing response Fig 3.

## Discussion

We have demonstrated that there are biologically feasible conditions by which MMT could arise. The resulting spatial distribution of fibroblasts is comparable with what we have observed in many NHP granulomas. This hypothesis is further supported with evidence that cells in the fibrotic cuff of granulomas that exhibit peripheral fibrosis have macrophage-like characteristics. These macrophage-like characteristics are known to be expressed on migratory cells and are not thought to be markers of fibroblasts since fibroblasts and myofibroblasts are thought to be tissue resident (i.e. not migratory) [48].

A better understanding of the processes that regulate development of fibrosis in TB has important implications for treatment and disease resolution. Fibrosis has been difficult to investigate in TB however because there is not a small animal model that recapitulates end-stage disease and human granulomas are not readily accessible for study. NHP granulomas replicate human TB, including the fibrotic outcomes associated with drug treatment and can serve as an alternative system where the basic immune mechanisms can be investigated. To address this problem, we characterized the phenotype of fibrotic NHP granulomas and used a systems biology approach to identify the basic biology of fibrosis in TB. Our work suggests that MMT may lead to peripheral fibrosis in granulomas, and although not well understood, MMT may underlie aspects of TB that have been difficult to explain up to this point. MMT has been demonstrated to occur *ex-vivo* when peripheral blood monocyte-derived macrophages are polarized to become M1 macrophages and given fluid from healing but not chronic wounds [38]. To date there has been limited research into MMT’s contribution to lung fibrosis. Using a computational approach, we demonstrated that MMT dynamics could be an important contributor to granuloma-associated fibrosis in the *Mtb* infected lung. Through complementary IHC staining of fibrotic granulomas from NHPs infected with TB 10–12 weeks after infection, we identified a population of CD11c+αSMA+ cells in the fibrotic region of granulomas suggesting the involvement of cells that are derived from sources other than lung resident fibroblasts.

Our results suggest that MMT occurs in macrophages that receive inflammatory (M1-polarizing) signals before receiving secondary anti-inflammatory signals. We also show that MMT in *GranSim* can be inhibited by exposure to *Mtb* and by parameters that drive inflammation e.g. the recruitment rate of IFNγ-producing T cells and the rate at which TGFβ downregulates inflammatory cytokine production. It is important to note that our computational model includes only STAT3 as an abstraction for all anti-inflammatory regulators, thus while *GranSim* predicts both STAT1 and STAT3 are involved in MMT, these pathways are actually representative of any alternative anti-inflammatory pathway with similar dynamics to the STAT3/IL-10 pathway and a combination of these pathways could be responsible for driving MMT *in vivo*, and further experimentation is needed to fully explore this hypothesis. This has been previously reported for pSMAD3/TGF-β signaling and MMT in a murine model of renal fibrosis [49] and cannot be discounted considering the expression patterns of these molecules in NHP granulomas [6]. It remains unknown if these mechanisms operate independently or in tandem in granulomas, but results and *in vitro* studies identifying M1 cells as sources of MMT in biology [38,49,50] suggest the STAT1/STAT3 axis is a significant contributor. We can compare our results observed in this study with a previously developed model exploring the involvement of lung-resident fibroblasts in granuloma associated fibrosis [21]. The distinct phenotypic diversity in TB granulomas has been difficult to recapitulate with lung fibroblasts alone but when we compare the virtual granulomas developed by our MMT-including model with the virtual granulomas produced by Warsisnke *et al* (sample shown in S2 Fig), we find that the updated model produces virtual granulomas that are more phenotypically similar to biologic granulomas with peripheral, or central fibrosis. Moreover, our virtual granuloma snapshots compare favorably with the fibrotic granulomas from antibiotic-treated animals [6], further supporting our hypothesis that MMT can generate multiple and biologically relevant phenotypes of granuloma-associated fibrosis.

The transformation of macrophages into myofibroblasts has important implications for granuloma function, interaction between networks of cells within the granuloma, and granuloma-scale immunity. When protective, this process may occur in a way that gradually increases fibrosis and serves to contain bacterial populations. However, this would occur in combination with persistent, but gradually decreasing, macrophage antimicrobial function. This would be accompanied by increasing levels of stiffness and extracellular matrix deposition and restricted diffusion of chemotactic ligands out of Mtb-rich regions coupled with limited migration of new immune cells into these areas. This approach may be preferable to a rapid influx of conventional fibroblasts that rapidly attenuate macrophage antimicrobial activity or produces a difficult-to-resolve scar that leads to permanent loss of lung function. Alternatively, inappropriately timed or positioned macrophage to fibroblast transition could limit antibacterial activity by restricting T cell access to Mtb-infected macrophages and decrease bacterial exposure to macrophage-expressed antimicrobial factors. This could lead to liquefaction of the granuloma and potential dissemination of bacteria.

We demonstrate a potential role for macrophages in development of granuloma-associated fibrosis and our work provides a basis for further *in vivo* investigation for the role of myeloid cells in fibrotic lung disease, including idiopathic pulmonary fibrosis. Reducing granuloma associated fibrosis could improve lung function and shorten TB treatment by improving the ability of antimycobacterial drugs to access difficult-to-target microenvironments where mycobacteria exist. Alternatively, there is the possibility that inhibiting fibrosis could increase dissemination of bacteria and hinder their clearance, so more work is needed to understand the trade-off of protective versus pathologic roles that fibrosis plays in TB. Moreover, additional work investigating how collagen is deposited in the granuloma needs to be performed before the impact of reducing fibrosis on disease burden can be predicted. The role of collagen deposition in the granuloma is not well characterized *in vivo* and further work in this area is required to fully understand the impact of reducing granuloma fibrosis, particularly since the conservation of immune balance is particularly pertinent in TB [51].

## Supporting information

S1 Fig Location of cell types after 150 days of simulated time for a granuloma exhibiting peripheral fibrosis. (TIFF)

S2 Fig Two samples of snapshots from fibrotic granulomas in the model with only lung resident fibroblasts. (TIFF)
