# Supplementary figures and images for: Systems biology predicts that fibrosis in tuberculous granulomas may arise through macrophage-to-myofibroblast transformation
Source: PLoS Comput Biol. 2020 Dec 28;16(12):e1008520. doi: 10.1371/journal.pcbi.1008520 (PMC7793262; doi:10.1371/journal.pcbi.1008520)

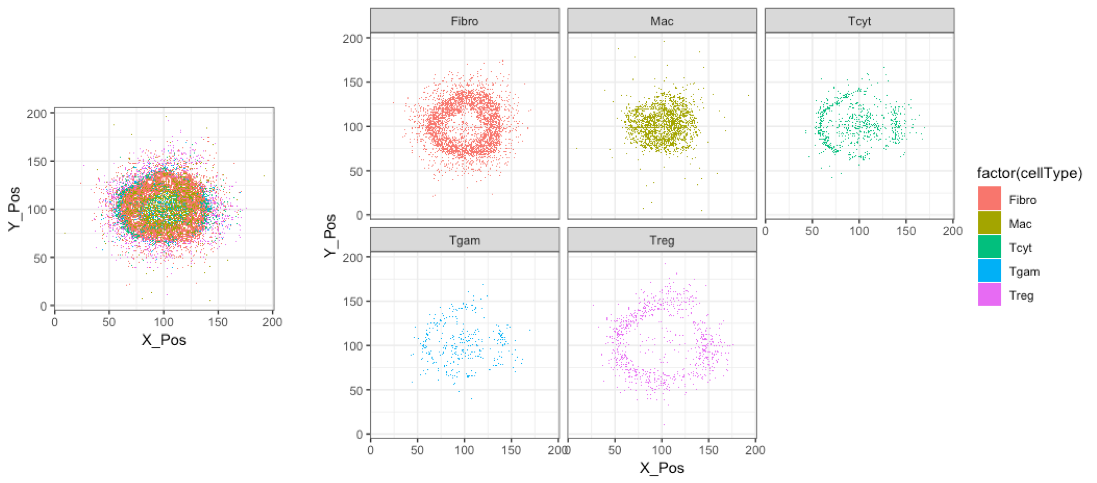

Supplement: S1 Fig — (TIFF) [file pcbi.1008520.s001.tiff]

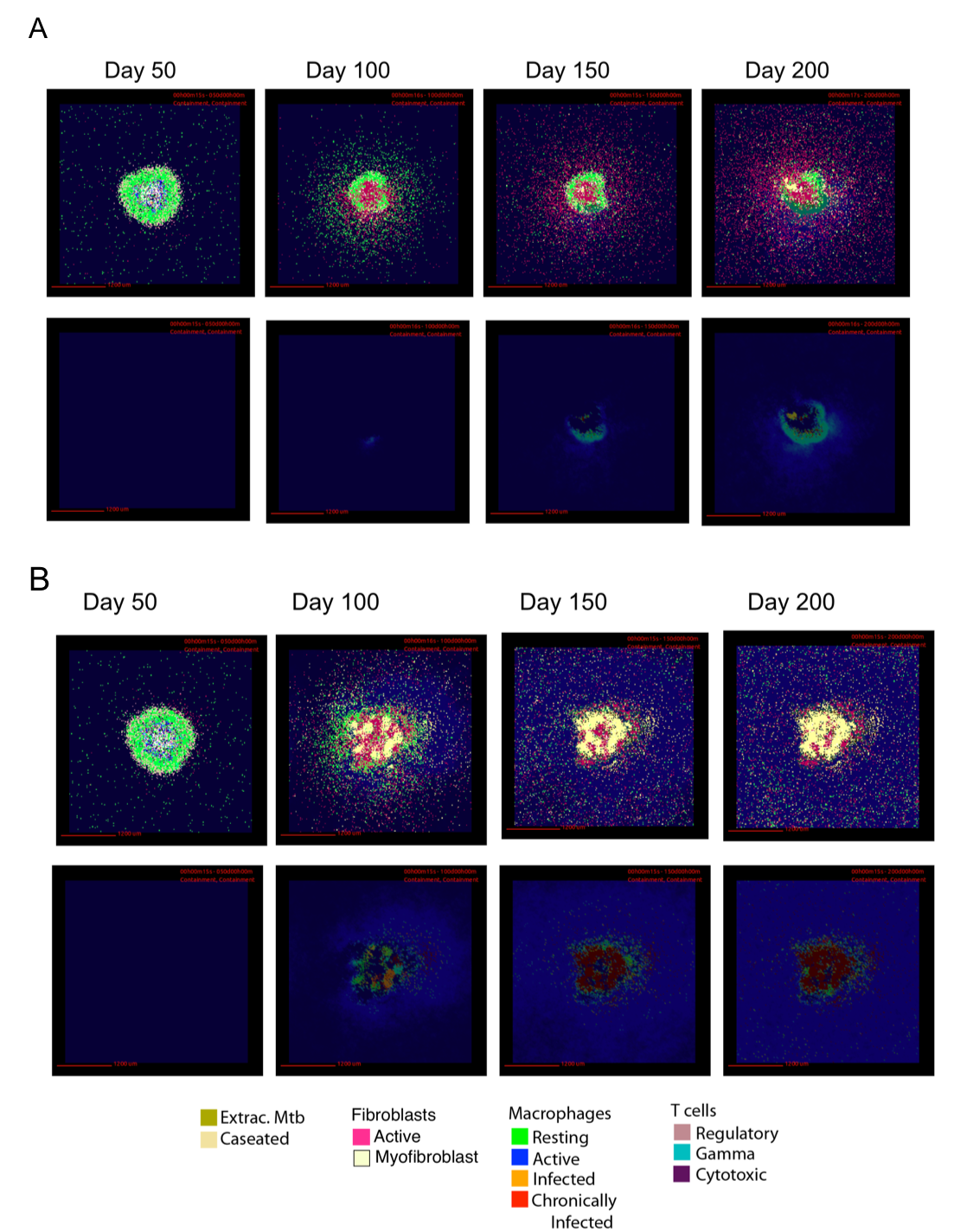

Supplement: S2 Fig — (TIFF) [file pcbi.1008520.s002.tiff]
